# Oral administration of Chinese herbal medicine during gestation period for preventing hemolytic disease of the newborn due to ABO incompatibility: A systematic review of randomized controlled trials

**DOI:** 10.1371/journal.pone.0180746

**Published:** 2017-07-18

**Authors:** Huijuan Cao, Ruohan Wu, Mei Han, Patrina Ha Yuen Caldwell, Jian-Ping Liu

**Affiliations:** 1 College of Traditional Chinese Medicine, Beijing University of Chinese Medicine, Beijing, China; 2 Centre for Kidney Research & Discipline of Paediatrics and Child Health, The Children’s Hospital at Westmead and Discipline of Paediatrics and Child Health, University of Sydney, Sydney, Australia; Fudan University, CHINA

## Abstract

**Background:**

About 85.3% of hemolytic disease of the newborn (HDN) is caused by maternal-fetal ABO blood group incompatibility. However, there is currently no recommended “best” therapy for ABO incompatibility during pregnancy.

**Objectives:**

To systematically assess the safety and effectiveness of oral Chinese herbal medicine (CHM) for preventing HDN due to ABO incompatibility.

**Methods:**

The protocol of this review was registered on the PROSPERO website (No. CRD42016038637).Six databases were searched from inception to April 2016. Randomized controlled trials (RCTs) of CHM for maternal-fetal ABO incompatibility were included. The primary outcome was incidence of HDN. The Cochrane risk of bias tool was used to assess the methodological quality of included trials. Risk ratios (RR) and mean differences with 95% confidence interval were used as effect measures. Meta-analyses using Revman 5.3 software were conducted if there were sufficient trials without obvious clinical or statistical heterogeneity available.

**Results:**

Totally 28 RCTs involving3413 women were included in the review. The majority of the trials had unclear or high risk of bias. Our study found that the rate of HDN and the incidence of neonatal jaundice might be 70% lower in the herbal medicine group compared with the usual care group (RR from 0.25 to 0.30).After treatment with herbal medicine, women were twice as likely to have antibody titers lower than 1:64 compared with women who received usual care(RR from 2.15 to 3.14) and the umbilical cord blood bilirubin level in the herbal medicine group was 4umol/L lower than in those receiving usual care. There was no difference in Apgar scores or birthweights between the two groups.

**Conclusions:**

This review found very low-quality evidence that CHM prevented HDN caused by maternal-fetal ABO incompatibility. No firm conclusions can be drawn regarding the effectiveness or safety of CHM for this condition.

## Introduction

Maternal-fetal incompatibility is one of the most common immunological causes for spontaneous abortions, with 15% being related to maternal-fetal ABO incompatibility [1]. About 85.3% of hemolytic disease of newborn (HDN) is also caused by maternal-fetal ABO incompatibility [2], which may result in neonatal jaundice. It is estimated that27% of newborns in China have ABO incompatibility compared with 15% worldwide [3]. During pregnancy, there are often no symptoms in the pregnant woman with ABO incompatibility, but 30% of babies born to these women have HDN, with 19% having moderately severe to severe hemolysis [4].

Intrauterine therapy such as maternal plasma exchange or intrauterine fetal blood transfusions are accepted therapies during pregnancy for maternal-fetal Rh incompatibility, as this condition has a higher risk of morbidity and mortality. Even though maternal plasma exchange is relatively safe, the procedure can be difficult and expensive [5]. On the other hand, intrauterine fetal blood transfusions have the potential risk for preterm pre-labor rupture of the membranes[6] (PPROM, 0.1%-2% risk), intra-amniotic infection (chorioamnionitis, 0.3%-1.2% risk), preterm labor, and other fetal complications (1.3%-2.5% risk).There is currently no recommended “best” therapy for preventing ABO incompatibility during pregnancy. In China intravenous injections of glucose and vitamin C in combination with oral Vitamin E are the commonly used for this condition.

ABO blood group incompatibility is recorded as *Taihuang* (fetal jaundice) or *Tailou* (threatened abortion) according to traditional Chinese medicine (TCM) diagnosis. In TCM theory, syndrome differentiation for this disease often referred to retained dampness-heat stagnation [7] and kidney-qi deficiency [8]. Consequently, clearing heat and draining dampness, warming the kidney and fortifying the spleen are the general treatment principles for this condition [9]. Previous studies published in China indicated that Herba Artemisiae Scopariae (Yinchen) and Rhizoma Rhei (Da Huang) have high contents of blood group A and B substances, which may resist antibodies from maternal-fetal incompatibility [10]. 6,7-dimethoxy coumarin, one of the ingredients of Herba Artemisiae Scopariae (Yinchen) is thought to protect the liver [11] and cause jaundice resolution [12]. A previous systematic review indicated a potential benefit for using combined therapy of Chinese and Western medicine to treat maternal-fetal ABO incompatibility [1]. However, due to the poor methodological quality of the included studies and obvious clinical heterogeneity between the trials, the study could not draw firm conclusions.

## Objectives

To critically appraise the existing randomized controlled trials (RCTs) of Chinese herbal medicine (CHM) for treating ABO incompatibility during pregnancy to prevent hemolytic disease of the newborn, and provide evidence-based evaluation of the safety and effectiveness of the oral CHM.

## Methods

The protocol of this review was registered on the PROSPERO website (No. CRD42016038637) and can be retrieved through https://www.crd.york.ac.uk/PROSPERO/display_record.asp?ID=CRD42016038637 (see S1 File).This review was conducted and reported according to the Preferred Reporting Items for Systematic Reviews and Meta-Analyses (PRISMA) statement guidelines (see S2 File).

### Criteria for considering studies for this review

Randomized controlled trials (RCTs) which compared oral administration of CHM (alone or in combination with other treatments) with other therapies (or no treatment)for treating maternal-fetal ABO incompatibility were included in our review. We defined ABO incompatibility as the situation when the maternal blood type is "O", and the paternal blood type is A, B or AB resulting in an antibody titer (IgG anti-A or anti-B) that is higher than 1:64. The primary outcome was the incidence of HDN; secondary outcomes of interest were: the level of antibody titer after treatment, the incidence of neonatal jaundice, neonatal bilirubin levels (including neonatal umbilical cord blood bilirubin and total serum bilirubin), other measurements of health status of the newborn (such as Apgar scores and birthweight) and adverse events. To be included in our evaluation, at least one of the above outcomes need to be reported in the trial.

### Search methods for identification of studies

Two English databases and four Chinese databases were searched from inception to April 2016, including the Cochrane Central Register of Controlled Trials (CENTRAL), PubMED, China Network Knowledge Infrastructure(CNKI), Chinese Scientific Journals Database (VIP), WanFang Database (for unpublished graduate theses in China), and Chinese Biomedicine (CBM). Details of the search strategies are shown as below.

#1: "Medicine, Chinese Traditional" [Mesh]; #2: "Drugs, Chinese Herbal" [Mesh]; #3: "ABO incompatibility" [Mesh]; #4: “ABO hemolytic disease of newborn” [Mesh]; #5: (#1 OR #2) AND (#3 OR #4)

The above strategies were adapted for each specific database, with the use of Chinese characters for relevant key words when searching Chinese databases.

### Study selection

Two review authors (Cao H and Wu R) independently assessed the titles, abstracts and keywords of every record retrieved to determine relevance and whether the study was an RCT according to the inclusion criteria. Full articles were retrieved for further assessment, with reasons for exclusion being recorded. Where differences in opinions existed, they were resolved by discussion with the other authors until a consensus was reached.

### Data extraction and management

Data concerning details of the included studies were extracted independently by two review authors (Cao H and Wu R) using a piloted data extraction form. The data extraction form included general information, study design, participant information, interventions and outcomes. Authors of relevant studies identified were contacted if needed to obtain information regarding additional references, unpublished trials, or data missing from the original publication. Disagreements were resolved by consensus.

### Assessment of risk of bias in included studies

Two authors (Cao H and Han M) assess the methodological quality of the included trials independently. Selection bias (random sequence generation and allocation concealment), detection bias (blinding of outcome assessment), attrition bias (incomplete outcome data), reporting bias (selective reporting), and other biases (including sample size calculation, inclusion/exclusion criteria for patients' recruitment, comparability of baseline data, funding sources, and any other potential methodological flaws that may have influenced the overall results) were assessed according to the criteria from the Cochrane Handbook for Systematic Reviews of Intervention [13]. Since all of the outcomes of this review were measured objectively and were therefore unlikely to be influenced by the lack of blinding, and there was no ideal placebo control for Chinese herbal medicine (especially for the herbal decoction), we did not assess for performance bias (with blinding of participants and personnel). The level of bias was assessed for each trial (as low, high or unclear risk). A study was considered to have a low risk of bias if all seven risk of bias items met the criteria as “low risk”, a study was considered to have high risk of bias if at least one of the seven items was assessed as “high risk”.

### Data analysis

Data were summarized using risk ratios (RR) with 95% confidence intervals (CI) for binary outcomes or mean difference (MD) with 95% CI for continuous outcomes. We used Revman 5.3 software from the Cochrane Collaboration for data analyses. Meta-analysis was used if there was acceptable homogeneity in the study design, participants, interventions, control, and outcome measures. Statistical heterogeneity was tested by examining *I*^*2*^ [14], with an *I*^*2*^greater than 50% indicating a possibility of statistical heterogeneity. Both fixed effects models and random effects models were used if there was a possibility of statistical heterogeneity between trials. Pooling analysis were not performed if there was large statistical heterogeneity (*I*^*2*^>75%). Publication bias was explored by funnel plot analysis. Subgroup analyses were conducted for different baseline characteristics or different treatment duration if data were sufficient. Sensitivity analyses were used to determine whether the conclusions differed if eligibility was restricted to studies without high risk of bias, or if a fixed effect/random effect model had been applied.

## Results

### Results of the search

**Fig 1:**
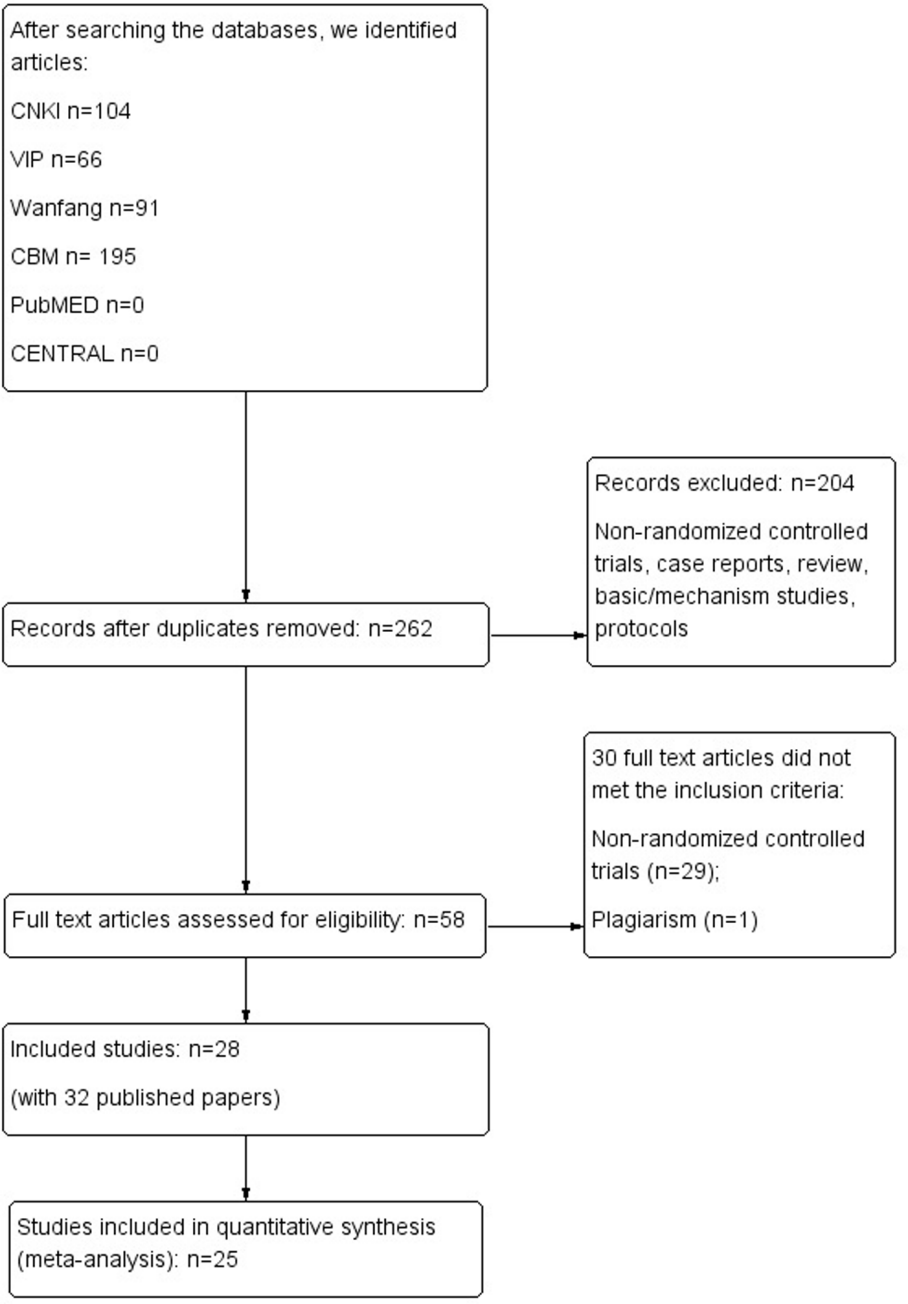
Study flow chart.

A totally of 262 papers were identified through literature searching, and 58 full text papers were retrieved for further assessment. Finally, 32 publications of 28trials [15–46] met our pre-defined criteria and were included in this review. There were four duplicate studies [16&^17^, ^31^&^32^, ^33^&^34^, ^35^&^36^]. One paper [47] was excluded due to plagiarism. All of the included trials were conducted and published in China. Details of the searching and selection of included studies are shown in Fig 1.

### Characteristics of the included studies

The 28 trials involved 3413 women, with an average of 57 women in each group. The average age of participants was 27.7 years, and the baseline gestational was around 25.0 weeks. Six of the trials [20, 27–29, 39, 41] enrolled pregnant women whose antibody titers were higher than 1:64 at baseline, and the remaining trials included women with antibody titers higher than 1:128.

Twenty-seven of the 28 included trials assessed the use of oral CHM for preventing hemolytic disease of the newborn due to ABO incompatibility, with 22 trials [15, 16, 18–20, 22–26, 29–33, 37, 39, 41, 43–46] using herbal decoctions, four [21, 27, 28, 40] using herbal liquid (patent) and one trial [36] using herbal granule. The remaining trial [42] assessed the use of oral administration and external washing of CHM. According to TCM theory, all of the included trials used clearing heat and draining dampness as their treatment principle. The top five most frequently used herbs were Herba Artemisiae Scopariae (Yin Chen, in 26 trials), Rhizoma Glycyrrhizae (Gan Cao, in 23 trials), Fructus Gardeniae (Zhi Zi, in 22 trials), Radix Scutellariae (Huang Qin, in 22 trials), and Radix et Rhizoma Rhei (Da Huang, in 22 trials). Except Rhizoma Glycyrrhizae, which was used for coordinating the drug actions of a prescription, the other four herbs were all used for clearing heat. Herba Artemisiae Scopariae and Fructus Gardeniae also works on draining dampness.

Twenty-three of the included trials compared herbal decoction with usual care, four trials [16, 21, 24, 28] assessed herbal decoction as an add-on treatment combined with usual care compared to usual care alone, and one trial [41] compared herbal decoction with no treatment. Usual care included vitamin supplements(in all trials: oral or intravenous vitamin C at 100-200mg three times daily and oral vitamin B6 at 20mg twice or three times daily), oxygen inhalation (in 18 trials, for 20-30minutes once daily) and oral phenobarbital (in 10 trials: at 20-30mg twice or three times daily for two to four weeks before delivery).

The treatment duration varied among the trials. Participants in five trials [19, 21, 22, 40, 44] received treatments for ten days during pregnancy. Treatment duration in 18 trials [18, 23, 24, 26, 27, 29–31, 33, 36–39, 41–43, 45, 46] was for at least two to six weeks depending on the level of the antibody titer. One trial [25] reported that the treatment was continued until labor. And four trials [15, 16, 20, 28] did not mention the length of treatment.

For the other outcomes, HDN was reported in 15 trials [16, 19, 20, 22, 23, 25, 26, 29–31, 33, 36, 41, 42, 46], the level of antibody titer after treatment was reported in 25 trials [18–31, 33, 36–40, 42–46], the incidence of neonatal jaundice was reported in 13 trials [15, 16, 18–20, 24–27, 29, 30, 36, 37], umbilical cord blood bilirubin was reported in seven trials [16, 18–20, 26, 36, 38], Apgar scores were reported in eight trials [18–21, 23, 26, 36, 40], birthweight was reported in six trials [18, 19, 21, 23, 36, 40], and adverse events were mentioned in eight trials [16, 21, 23, 30, 37, 38, 41, 42].

Details of the characteristics of the 28 included trials are shown in Table 1, and the components of the herbal prescriptions and adverse events are shown in Table 2.

**Table 1:** Characteristics of 28 included trials concerned Chinese herbal medicine for ABO incompatibility.

Study ID	Number of the participants	Antibody titer at baseline	Age (yrs)	Gestational weeks	Intervention	Control	Outcome measurements
An 2014a [15]	29:29	>1:128	T:30.8±11.3 C:31.4±10.7	T:18.4±4.9 C:16.3±3.7	Huangyin Antai Decoction, methods for administration were not reported	Vit C 100mg Tid, Vit E 100mg Tid, oxygen inhalation	Time for antibody titer less than 1:64, NJ
An 2014b [16]	30:30	>1:128	T:23.5±6.1 C:22.1±5.3	T:17.5±5.3 C:18.2±6.1	Huangyin Antai Decoction plus control therapies, methods for administration were not reported	Vit C 100mg Tid, Vit E 100mg Tid, oxygen inhalation	NJ, HDN, UCBB, change of antibody titer, adverse events
Cai 2009 [18]	30:30	>1:128	T:28.4±4.1 C:27.6±3.2	T:27.1±6.3 C:25.4±5.5	Lianhuang Decoction 250ml Bid for 2 weeks	Vit C 2g, Vit B6 0.2g intravenous injection, Vit E 100mg Bid, oxygen inhalation 30min once daily for 2 weeks. Phenobarbital30mg Bid for 10 days after 36 or 37 gestational weeks.	Apgar scores, NJ, Number with antibody titers lower than 1:64 post treatment, UCBB, birthweight
Chen 2011 [19]	32:25:28	>1:128	27.5±6.5	>16	T1: Gutai Yinchen Decoction 200ml Bid; T2: Yinchenhao Decoction 200ml Bid; Oral administration for 10 days at 26, 30, 34 gestational weeks	Vit C 100mg Tid, Vit E 50mg Tid, oral administration for 10 days at 26, 30, 34 gestational weeks. Pheonbarbital was given 30mg Bid for 10 days after 36 gestational weeks.	Apgar scores, NJ, HDN, number with antibody titers lower than 1:128 post treatment, UCBB, birthweight, adverse events
Cui 2014 [20]	57:58	>1:64	T: 28.2±4.1 C:27.0±3.3	T:26.9±5.8 C:25.3±4.8	Baishao Gancao Decoction plus Yinchen Dazao Decoction, methods for administration were not reported	Vit C 0.2g Tid, Vit B6 20mg Tid, Vit E 50mg Bid, oxygen inhalation 30min daily for 10 days as a course of treatment. Phenobarbital30mg Bid for 10 days after 36 gestational weeks.	Apgar scores, NJ, HDN, number with antibody titers lower than 1:64 post treatment, UCBB
Ding 2002 [21]	57:42	>1:128	Not reported	>16	Kangtuihuang Oral Liquid 10ml Bid plus the control therapy. The treatment was given 10 days every 4 weeks.	Vit C 1g intravenous injection, Vit E 100mg Bid, oxygen inhalation 20min Bid. The treatment was given 10 days every 4 weeks.	Change in antibody titers, Apgar scores, birthweight, time and severity of the neonatal jaundice, 96 hour total serum bilirubin
Feng 2006 [22]	148:140:123	>1:128	T1:27.5±3.8 T2:27.5±3.9 C:26.3±3.6	T1:26±5.5 T2:27.5±5.2 C25.8±5.1	T1: Decoction of Herba Arib (100g) 250ml daily; T2: Jigucao Decoction 250ml daily. The treatment was given 10 days every 4 weeks.	Vit C 0.2g Tid, Vit B6 20mg Tid, Vit E 50mg Bid, oxygen inhalation 30min daily. Oral administration for 10 days at 24, 30, 33 gestational weeks. Phenobarbital 30mg Bid for 10 days after 36 gestational weeks.	HDN, number with antibody titers lower than 1:64 post treatment
Hu 2014 [23]	91:90:87	>1:128	20–40	26–34	T1: Quhuang Antai Decoction 200ml Bid; T2: Yinchenhao Decoction 200ml Bid. Treatment was given at least 28 days	Vit C 100mg Tid, Vit E 100mg Bid, oxygen inhalation 30min daily for 10 days every 4 weeks. Phenobarbital20-30mg Tid after 36 gestational weeks. Treatments were given at least 28 days	Apgar scores, time of the neonatal jaundice, HDN, number with antibody titers lower than 1:128 post treatment, birthweight, adverse events
Jin 2013 [24]	30:30	>1:128	22–40, average 27.6	13–36	Self made prescription once daily for 4 weeks, plus the control therapy	Vit C100mg Tid, Vit E 100mg Tid. The treatment was given 10 days every 4 weeks.	NJ, number with antibody titers lower than 1:128 post treatment
Li 2013 [25]	20:20	>1:128	23–43	Unclear	Modified Yinchenhao Decoction 200ml Bid. The treatment last until labor	Vit C 100mg Tid, Vit E 100mg Bid, oxygen inhalation 30min daily. Oral administration for 10 days at 28, 32, 36 gestational weeks. Phenobarbital 20mg Tid after 36 gestational weeks.	NJ, number with antibody titers lower than 1:128 post treatment, HDN
Li 2014 [26]	28:28	>1:128	T:30.8±9 C:29.7±8.7	T:17.9±4.5 C:16.9±3.7	Modified Huangyin Antai Decoction Tid for 30 days	Vit E 100mg Tid, Vit C 100mg Tid, oxygen inhalation daily.	Apgar scores, NJ, HDN, number with antibody titers lower than 1:64 post treatment, UCBB, adverse events.
Li 2015 [27]	45:45	>1:64	T:29.3±8.7 C:28.7±7.6	T:21.6±7.3 C:22.4±6.3	Yinzhihuang Oral Liquid 10ml Tid for at least 4 weeks	Vit C 2g intravenous injection, oxygen inhalation 30min daily for 4 weeks	NJ, number with antibody titers lower than 1:64 post treatment
Liu 2015 [28]	42:42	>1:64	23–39, average 25.6	14–36	Yinzhihuang Oral Liquid 20ml Tid for 4 weeks, plus control therapy	Vit C 3g intravenous injection, Vit E 100mg Bid, for 10 days at 20, 34, 28 gestational weeks.	Time of neonatal jaundice, number with antibody titers lower than 1:64 post treatment, serum total bilirubin
Luo 2013 [29]	50:50	>1:64	26.8±3.1	12–33	Herbal Decoction once daily for 14 days	Vit C 1g intravenous injection, Vit E 100mg Bid, oxygen inhalation 30min daily for 14 days	HDN, NJ, Number with antibody titers lower than 1:64 or 1:128 post treatments.
Lv 2012 [30]	30:30	>1:128	T:28.17±5.15 C:28.03±5.25	>12	Yinchen Erdan Erhuang Decoction Tid for at least 4 weeks	Vit E 100mg Tid, Vit C 100mg Tid, oxygen inhalation 30min daily for at least 4 weeks	Number with antibody titers lower than 1:64/1:128 post treatments, HDN, NJ, time for taking medicine, adverse events
Mei 2006 [31]	54:42	>1:128	21–38	Not reported	Yinqi Decoction once daily for 28 days per course, 1–2 courses	Vit E 100mg Tid, Vit C 100mg Tid, oxygen inhalation 30min daily. The treatment was given 10 days every 4 weeks.	HDN, Number with antibody titers lower than 1:64 post treatments.
Su 2005 [33]	70:70:70	>1:128	T1:27.4±3.5 T2:27.6±3.8 C:27.3±3.7	T1:26.1±5.6 T2:25.8±5.3 C:25.4±5.5	T1: Jigucao Decoction for 20–30 days; T2: Fufang Yinchen Decoction for 20–30 days	Vit C 0.2g Tid, Vit B6 20mg Tid, Vit E 50mg Bid, for 20–30 days. Oxygen inhalation 30min daily for 10 days at 24, 30, 33 gestational weeks. Pheonbarbital30mg Bid for 10 days after 36 or 37 gestational weeks.	HDN, Number with antibody titers lower than 1:64 or 1:128 post treatments.
Sun 2008 [36]	94:78	>1:128	26.78±3.07	12–33	Yinzhi Kangrong Granules Bid for 7–14 days	Vit C 1g intravenous injection, folic acid 5mg Tid, Vit B6 20mg Tid, Vit E 100mg Bid, oxygen inhalation 30min daily. Oral administration for 10 days at 24, 30, 33 gestational weeks. Pheonbarbital30mg Bid for 10 days after 39 gestational weeks.	Apgar scores, NJ, HDN, Number with antibody titers lower than 1:64 post treatment, UCBB, hemoglobin, birthweight, adverse events.
Xu B 2011 [37]	83:83	>1:128	T:27.6±3.1 C:28.1±3.2	T:25.2±2.4 C:24.8±2.4	Modified Yinchenhao Decoction 200ml Bid. Pheonbarbital20-30mg Bid for 7 days after 36 gestational weeks.	Vit C 0.5g intravenous injection, Vit E 100mg Bid. Pheonbarbital 20-30mg Bid for 7 days after 36 gestational weeks.	NJ, Number with antibody titers lower than 1:64 post treatment, serum total bilirubin, adverse events.
Xu J 2009 [38]	80:70	>1:128	T:28.8 C:26.5	Not reported	Fangrong Antai Power 100ml Bid for at least 2 weeks	Vit C 1g intravenous injection, Vit E 100mg Bid for at least 2 weeks	Number with antibody titers lower than 1:128 post treatment, UCBB, adverse events
Xu L 2011 [39]	30:30	>1:64	T:27.1 C:26.2	T:22.6 C:23.1	Taier Yixue Decoction 200ml Bid for at least 4 weeks	Vit C 1g intravenous injection, Vit E 100mg Bid, oxygen inhalation 20min Bid. Pheonbarbital20-30mg Tid after 38 gestational weeks.	Number with antibody titers lower than 1:64 post treatment
Yang 2015 [40]	64:56:50	>1:128	T1: 27.5 T2:25.9 C:25.6	T1:28.2 T2:28.8 C:29.8	T1: Yinzhihuang Oral Liquid 20ml Tid; T2: Yinchenhao Decoction 50-200ml Bid. Treatment was given 10 days at 26, 30, 34 gestational weeks.	Vit C 2g intravenous injection, Vit E 100mg once daily, 10 days at 26, 30, 34 gestational weeks.	Number with antibody titers lower than 1:128 post treatment, Apgar scores, birthweight
Yu 2013 [41]	92:90	>1:64	28.3±6.7	16–36	Self made prescription 3–4 times daily for 20–30 days, then once every two days	No treatment	Incidence, time and serious of the neonatal jaundice, HDN, adverse events
Zhang 2013 [42]	60:60	>1:128	20–39, average 27.6	16–36	Modified Yinchenhao Decoction 200ml Bid for 2 weeks, plus herbal decoction external used once daily	Vit C 100mg Tid, Vit E 100mg Bid for 2 weeks	Number with antibody titers lower than 1:64 post treatment, Incidence of the neonatal jaundice, HDN, adverse events
Zhao 2012 [43]	59:52:57	>1:128	T1:27.33±6.03 T2:27.14±6.12 C:27.52±6.43	Not reported	T1: Xiaohuang Decoction 200ml Bid; T2: Yinchenhao Decoction 200ml Bid. At least 4 weeks treatment.	Vit C 1g intravenous injection, Vit E 100mg Bid, oxygen inhalation 30min once daily. At least 4 weeks treatment.	Number with antibody titers lower than 1:128 post treatment
Zhou 2007 [44]	41:36	>1:128	T:26.3±2.8 C:27.4±3.2	T:24.1±1.8 C:24.5±2.1	Modified Yinchenhao Decoction Bid for 10 days, plus control therapy	Vit C 0.5g intravenous injection, Vit E 100mg Bid, oxygen inhalation 30min once daily for 10 days	Number with antibody titers lower than 1:64 post treatment
Zong 2012 [45]	32:28	>1:128	26–35	10–16	Modified Erzhi Dihuang Yinchen Decoction 200ml Bid for 4 weeks	Vit C 100mg Tid, Vit E 100mg Bid for 4 weeks	Number with antibody titers lower than 1:64 post treatment
Zou 2001 [46]	98:98	>1:128	Not reported	16–34	Yincan Erchen Decoction 200ml Bid for 2–6 weeks according to the baseline antibody titer	Vit C 0.2g Tid, Vit E 100mg Tid, oxygen inhalation 30min once daily for 2–6 weeks according to the baseline antibody titer	Number with antibody titers lower than 1:64 post treatment, HDN

T: Treatment group; C: Control group; Vit: Vitamin; Tid: Three times daily; Bid: Twice daily; NJ: incidence of the neonatal jaundice; HDN: incidence of the newborn hemolytic disease; UCBB: umbilical cord blood bilirubin

**Table 2:** Components and adverse events of the herbal prescriptions in 28 included trials.

Study ID	Herbal Medicine	Component of Prescription	Adverse events
An 2014a [15]	Yinchen Antai Decoction	Herba Artemisiae Scopariae 10g, Radix Scutellariae 10g, Radix Rehmanniae 10g, Radix et Rhizoma Salviae Miltiorrhizae 10g, Cortex Phellodendri Chinensis 6g, Cortex Moutan 10g, Fructus Gardeniae 10g, Radix et Rhizoma Glycyrrhizae 3g, Cortex Lycii 10g, Radix et Rhizoma Rhei 5g	Not report
An 2014b [16]	Yinchen Antai Decoction	Radix et Rhizoma Rhei 5g, Radix et Rhizoma Salviae Miltiorrhizae 10g, Cortex Phellodendri Chinensis 6g, Radix et Rhizoma Glycyrrhizae 3g, Cortex Lycii 10g, Radix Rehmanniae 10g, Cortex Moutan 10g, Radix Scutellariae 10g, Herba Artemisiae Scopariae 10g, Fructus Gardeniae 10g; Generalized itching combine with Radix Angelicae Dahuricae; Yellow complexion combine with Rhizoma Polygoni Cuspidati; Lumbago combine with Herba Taxilli; Abdominal pain combine with Radix Paeoniae Alba	abortion or stillbirth(1/4)
Cai 2009 [18]	Lianhuang Decoction	Receptaculum Nelumbinis 10g, Radix Astragali 15g, Radix et Rhizoma Rhei 6g, Herba Artemisiae Scopariae 20g, Cortex Eucommiae 20g, Radix Aucklandiae 6g, Rhizoma Atractylodis Macrocephalae 10g, Herba Agrimoniae 20g	Not report
Chen 2011 [19]	GutaiYinchen Decoction	Semen Cuscutae 15g, Radix Dipsaci 15g, Rhizoma Atractylodis Macrocephalae15g, Herba Artemisiae Scopariae 12g, Radix Angelicae Sinensis 12g, Radix Paeoniae Alba12g, Radix et Rhizoma Rhei 6g, Radix et Rhizoma Glycyrrhizae 6g, Radix Scutellariae 9g, Fructus Gardeniae 10g	Not report
Cui L 2014 [20]	Baishaogancao Decoction combine with Yinchen Dazao Decoction	Radix Paeoniae Alba 30g, Herba Artemisiae Scopariae 30g, Radix et Rhizoma Glycyrrhizae Praeparata cum Melle 10g, 5 Fructus Jujubae	Not report
Ding 2002 [21]	Xiaokang Dihuang oral liquid	Herba Artemisiae Scopariae, Radix Gardeniae, Radix et Rhizoma Rhei, Fructus Jujubae, Rhizoma Atractylodis Macrocephalae, Radix Scutellariae, Radix Dipsaci, Herba Taxilli	Adverse event was not found in herbal medicine group
Feng 2006 [22]	T1:Jigucao;T2:Jigucao Decoction	T1 HerbaAbri;T2 Herba Abri 30g, Radix seu Caulis Berberidis Gagnepainii 15g, Poria 15g,3 Receptaculum Nelumbinis, Radix et Rhizoma Glycyrrhizae 8g	Not report
Hu 2014 [23]	Quhuang Antai mixture	Semen Cuscutae 30g, Cortex Eucommiae 10g, Rhizoma Atractylodis Macrocephalae 10g, Fructus Corni 10g, Fructus Lycii 10g, Radix Codonopsis 24g, Radix Polygoni Multiflori Praeparata cum Succo Glycines Sotae 24g, Fructus Alpiniae Oxyphyllae 10g, Fructus Citri Sarcodactylis 10g, Poria 10g, Herba Artemisiae Scopariae 10g, Fructus Gardeniae Praeparatus 10g, Radix Scutellariae 15g, Radix Angelicae Sinensis 15g, Pericarpium Citri Reticulatae 6g, Radix et Rhizoma Glycyrrhizae 5g	Adverse event was not found in herbal medicine group
Jin 2013 [24]	Self made prescription	Herba Artemisiae Scopariae, Fructus Gardeniae, Radix Scutellariae, Radix Codonopsis, Radix et Rhizoma Glycyrrhizae et al. kidney deficiency syndrome combine with Radix Dipsaci, Herba Taxilli, Cortex Eucommiae; Spleen deficiency syndrome combine with Radix Astragali, Rhizoma Atractylodis Macrocephalae, Rhizoma Dioscoreae, Radix Aucklandiae, Semen Coicis, Poria; Abdominal pain combine with Radix Angelicae Sinensis, Radix Paeoniae Alba; Vagina bleeding combine with Nodus Nelumbinis Rhizomatis, Herba Agrimoniae; Middle and late pregnancy combine with Radix et Rhizoma Salviae Miltiorrhizae, Radix Paeoniae Rubra, Herba Leonuri	Not report
Li 2013 [25]	Modified Yinchenhao decoction	Herba Artemisiae Scopariae 9g, Fructus Gardeniae Praeparatus 6g, Radix et Rhizoma Rhei 4g, Radix Scutellariae 9g, Rhizoma Atractylodis Macrocephalae 15g, Poria 10g, Radix et Rhizoma Glycyrrhizae 6g	Not report
Li 2014 [26]	HuangyinAntai decoction	Radix et Rhizoma Glycyrrhizae 3g, Radix et Rhizoma Salviae Miltiorrhizae 10g, Radix Scutellariae 10g, Fructus Gardeniae 10g, Cortex PhellodendriChinensis 6g, Radix Rehmanniae 10g, Radix et Rhizoma Rhei 5g, Cortex Moutan 10g, Cortex Lycii 10g, Herba Artemisiae Scopariae 10g; Yellow complexion combine with Rhizoma Polygoni Cuspidati; Abdominal pain combine with Radix Paeoniae Alba; Generalized itching combine with Radix Angelicae Dahuricae, white peony root, Herba Schizonepetae	Not report
Li 2015 [27]	T1:Yinzhihuang oral liquid; T2:Yinzhihuang oral liquid combine with Danshen	T1: Herba Artemisiae Scopariae, Fructus Gardeniae, baicalin, Flos Lonicerae Japonicae; T2:HerbaArtemisiaeScopariae, Fructus Gardeniae, baicalin, FlosLonicerae Japonicae, Radix et Rhizoma Salviae Miltiorrhizae	Not report
Liu 2015 [28]	Yinzhihuang Oral Liquid	Herba Artemisiae Scopariae, Fructus Gardeniae, baicalin, Flos Lonicerae Japonicae	Not report
Luo 2013 [29]	Self made prescription	Herba Artemisiae Scopariae, Radix et Rhizoma Salviae Miltiorrhizae, Eucommia ulmoides oliv, Semen Cuscutae, Radix Paeoniae Alba, Radix Dipsaci, Radix Astragali, Fructus Gardeniae, Radix Scutellariae, Radix et Rhizoma Rhei, Radix et Rhizoma Glycyrrhizae	Not report
Lv 2012 [30]	YinchenErdanErhuang Decoction	Herba Artemisiae Scopariae 10g, Fructus Gardeniae 10g, Radix et Rhizoma Rhei 3-6g, Cortex Moutan 10g, Radix et Rhizoma Salviae Miltiorrhizae 10g, Radix Scutellariae 10g, Cortex Phellodendri Chinensis 6g, Radix Rehmanniae 10, Cortex Lycii 10g; Generalized itching combine with Radix Angelicae Dahuricae, white peony root, Herba Schizonepetae; Lumbago combine with Radix Dipsaci, Herba Taxilli; Abdominal pain combine with Radix Paeoniae Alba, Radix et Rhizoma Glycyrrhizae Praeparata cum Melle; Yellow complexion combine with Rhizoma Polygoni Cuspidati	Adverse event was not found in herbal medicine group
Mei 2004 [31]	Yinqi mixture	Radix Astragali, Rhizoma Atractylodis Macrocephalae, Radix Dipsaci, Radix Scutellariae, Herba Artemisiae Scopariae, Fructus Gardeniae	Not report
Su 2005 [33]	T1:Jigucao Decoction; T2:Modified Yinchen Decoction	T1:HerbaAbri 30g, Radix seu Caulis Berberidis Gagnepainii 15g, Poria 15g, 3 Receptaculum Nelumbinis, Radix et Rhizoma Glycyrrhizae 8g; T2:Herba Artemisiae Scopariae 9g, Radix Scutellariae 9g, Radix et Rhizoma Rhei 4.5g, Radix et Rhizoma Glycyrrhizae 6g	Not report
Sun2008 [36]	Yinzhi Kangrong Decoction	Herba Artemisiae Scopariae 15g, Semen Cuscutae 10g, Fructus Gardeniae 10g, Radix Scutellariae 10g, Rhizoma Atractylodis Macrocephalae 10g, Radix et Rhizoma Salviae Miltiorrhizae 10g, Poria 10g, Polyporus 10g, Radix et Rhizoma Glycyrrhizae 3g	Not report
Xu J 2009 [37]	Fangrong Antai powder	Herba Artemisiae Scopariae 15g, Herba Taxilli 10g, Radix Paeoniae Alba 10g, Rhizoma Atractylodis Macrocephalae 10g, Radix et Rhizoma Rhei 5g, Radix Scutellariae 10g, Radix et Rhizoma Glycyrrhizae 5g; Threatened abortion combine with Radix Rehmanniae Praeparata 15g, Radix Astragali 12g, Semen Cuscutae 10g; Dampness-heat syndrome combine with Fructus Gardeniae10g, Herba Taraxaci 12g	abortion or stillbirth(0/3)
Xu B 2011 [38]	Modified Yinchenhao Decoction	Herba ArtemisiaeScopariae 15g, Fructus Gardeniae 12g, Radix et Rhizoma Rhei 3g, Radix Scutellariae 12g, FlosLonicerae Japonicae 12g, Herba Lysimachiae 12g, Radix Astragali 12g, Radix Codonopsis 12g, Radix Boehmeriae 10g, Radix et Rhizoma Glycyrrhizae 6g; Spleen deficiency syndrome combine with Rhizoma Dioscoreae 15g, Rhizoma Atractylodis Macrocephalae 12g; Kidney deficiency syndrome combine with Herba Taxilli 12g, Radix Dipsaci 15g, Cortex Eucommiae 15g; Vagina bleeding combine with Herba Agrimoniae 12g, Carbonizing Radix Rehmanniae 10g; Diarrhea reduce or stop using Radix et Rhizoma Rhei	Adverse event was not found in herbal medicine group
Xu L 2011 [39]	TaierYixue Decoction	Radix Rehmanniae 20g, Herba Artemisiae Scopariae 15g, Fructus Gardeniae 15g, Radix et Rhizoma Rhei 10g, Radix Scutellariae 10g, Rhizoma Coptidis 6g, Herba Leonuri 6g, Radix Angelicae Sinensis 15g, Rhizoma Chuanxiong 15g, Radix Paeoniae Alba 30g, Herba Agrimoniae 6g, Radix Aucklandiae 6g, Radix et Rhizoma Ginseng 6g, Radix et Rhizoma Glycyrrhizae 6g	Not report
Yang 2015 [40]	T1:Yinzhihuang Oral Liquid;T2:Yinchenhao Decoction	T1: Herba Artemisiae Scopariae, Fructus Gardeniae, baicalin, Flos Lonicerae Japonicae; T2: Herba Artemisiae Scopariae 9g, Fructus Gardeniae Praeparatus 10g, Radix Scutellariae 9g, Radix et Rhizoma Glycyrrhizae 6g, Radix et Rhizoma Rhei 4.5g	Not report
Yu 2013 [41]	Self made prescription	Herba Leonuri 30g, Radix Codonopsis 20g, Radix Angelicae Sinensis 15g, Herba Artemisiae Scopariae 15g, Semen Cuscutae 15g, Semen Persicae 12g, Rhizoma Cyperi 12g, Radix Curcumae 12g, Radix Scutellariae 12g, Rhizoma Atractylodis Macrocephalae 12g, Rhizoma Chuanxiong 9g, Radix et Rhizoma Rhei 9g, Radix et Rhizoma Glycyrrhizae Praeparata cum Melle 6g	abortion or stillbirth(2/10)
Zhan2013 [42]	Modified Yinchenhao Decoction	ORAL Herba Artemisiae Scopariae 15g, Fructus Gardeniae 9g, Radix et Rhizoma Rhei 3g, Herba Artemisiae Annuae 9g, Radix Scutellariae 9g, Cortex Eucommiae 15g, Semen Cuscutae 15g, Radix Dipsaci 15g, Endothelium Corneum Gigeriae Galli 9g, Radix et Rhizoma Glycyrrhizae 9g; Thirsty combine with Rhizoma Phragmitis 15g, Radix Glehniae 15g; Torpid intake combine with Fructus Amomi 6g, Pericarpium Citri Reticulatae; Sloppy stool stop using Radix et Rhizoma Rhei; Lumbago combine with Cortex Eucommiae Washing Herba Artemisiae Scopariae 30g, Fructus Gardeniae 15g, Radix et Rhizoma Rhei 10g, Herba Artemisiae Annuae 10g	Adverse event was not found in herbal medicine group
Zhao 2012 [43]	T1: Xiaohuang Decoction T2:Yinchenhao Decoction	T1:HerbaArtemisiaeScopariae 10g, Fructus Gardeniae Praeparatus 10g, Radix et Rhizoma Rhei 5g, Radix Scutellariae 15g, Pericarpium Citri Reticulatae 10g, Herba Artemisiae Annuae 10g, Semen Cuscutae 30g, Cortex Eucommiae 20g, Rhizoma Atractylodis Macrocephalae 20g, Radix et Rhizoma Glycyrrhizae 12g; T2: Herba Artemisiae Scopariae 18g, Fructus Gardeniae Praeparatus 10g, Radix et Rhizoma Rhei 6g	Not report
Zhou 2007 [44]	Yinchen Decoction	Herba Artemisiae Scopariae 12g, Radix et Rhizoma Rhei 4g, Radix Scutellariae 9g, Radix et Rhizoma Glycyrrhizae 6g	Not report
Zong 2012 [45]	Modified Erzhi Dihuang Yinchen Decoction	Fructus Ligustri Lucidi 12g, Radix Scutellariae 12g, Rhizoma Alismatis 12g, Poria 12g, Herba Ecliptae 15g, Radix Rehmanniae Praeparata 15g, Semen Cuscutae 15g, Herba Artemisiae Scopariae 20g, Rhizoma Zingiberis Recens 6g, Radix et Rhizoma Glycyrrhizae 10g	Not report
Zou 2002 [46]	Yincan Erchen Decoction	Herba Artemisiae Scopariae, Faeces Bombycis, Sclerotium Poriae Pararadicis, Rhizoma Pinelliae Praeparatum, Pericarpium Citri Reticulatae	Not report

### Risk of bias of included studies

All of the included trials stated that the treatments were randomly assigned, however only five of them [16, 18, 19, 26, 30] were assessed as having low risk of selection bias because they reported that a random numbers table was used to generate random allocation. Five trials [21, 23, 31, 36, 43] had unequal numbers between the groups, with two of them [23, 43] reporting they excluded participants after randomization if the blood type of the newborn was ‘O’. These five trials were evaluated as having high risk of selection bias. The remaining seventeen trials did not provide sufficient information to judge their risk of selection bias. Furthermore, none of the included trials mentioned allocation concealment, thus all of the trials were evaluated to have unclear risk of bias regarding to this item.

Except for three trials [19, 22, 25] which employed a third party to measure treatment outcomes, the other twenty-five trials were assessed as having unclear risk of detection bias.

Two trials [30, 36] were evaluated as having low risk of attrition bias because they clearly explained the reasons for drop outs, with equal numbers of drop outs (1 or 2 cases) in each group which is unlikely to impact on the final results. Three trials were evaluated as having high risk of attrition bias: one trial [37] reported a few cases of drop outs due to high and unchanged antibody titers during treatment and two other trials [23, 43] excluded participants after randomization if the blood type of the new born were ‘O’.

Since the protocols of the trials were unavailable, we determined the risk of reporting bias according to whether the trial reported all the key outcomes (HDN and antibody titer after treatment). Therefore, sixteen trials [16, 18–20, 22, 23, 26–31, 33, 36, 38, 41] had low risk of reporting bias and five trials [15, 39, 43–45] had high risk of reporting bias.

Only one trial [30] was assessed as having low risk of other bias, as this trial was well designed with an appropriate sample size calculation. Two trials [21, 24] had only one author and another two trials [28, 43] had poor methodology which may influence the results. These four trials were defined as having high risk of other bias.

**Fig 2:**
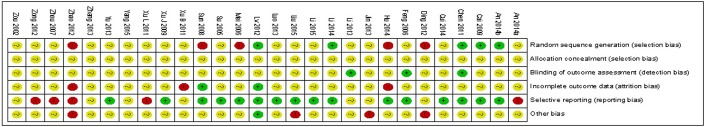
Risk of bias summary for each trial.

In summary, twelve of the included trials [15, 21, 23, 31, 36, 37, 38, 43–45] had poor methodological quality due to the potential high risk of selection, attrition, reporting or other bias. Only five trials [16, 18, 19, 26, 30] were assessed as having low risk of bias because of adequate randomization and completeness of the report. The remaining trials all had unclear risk of bias. The risk of bias summary for each trial is showed in Fig 2.

### Effects of interventions

#### Primary outcomes

Hemolytic Disease of the Newborn

**Fig 3:**
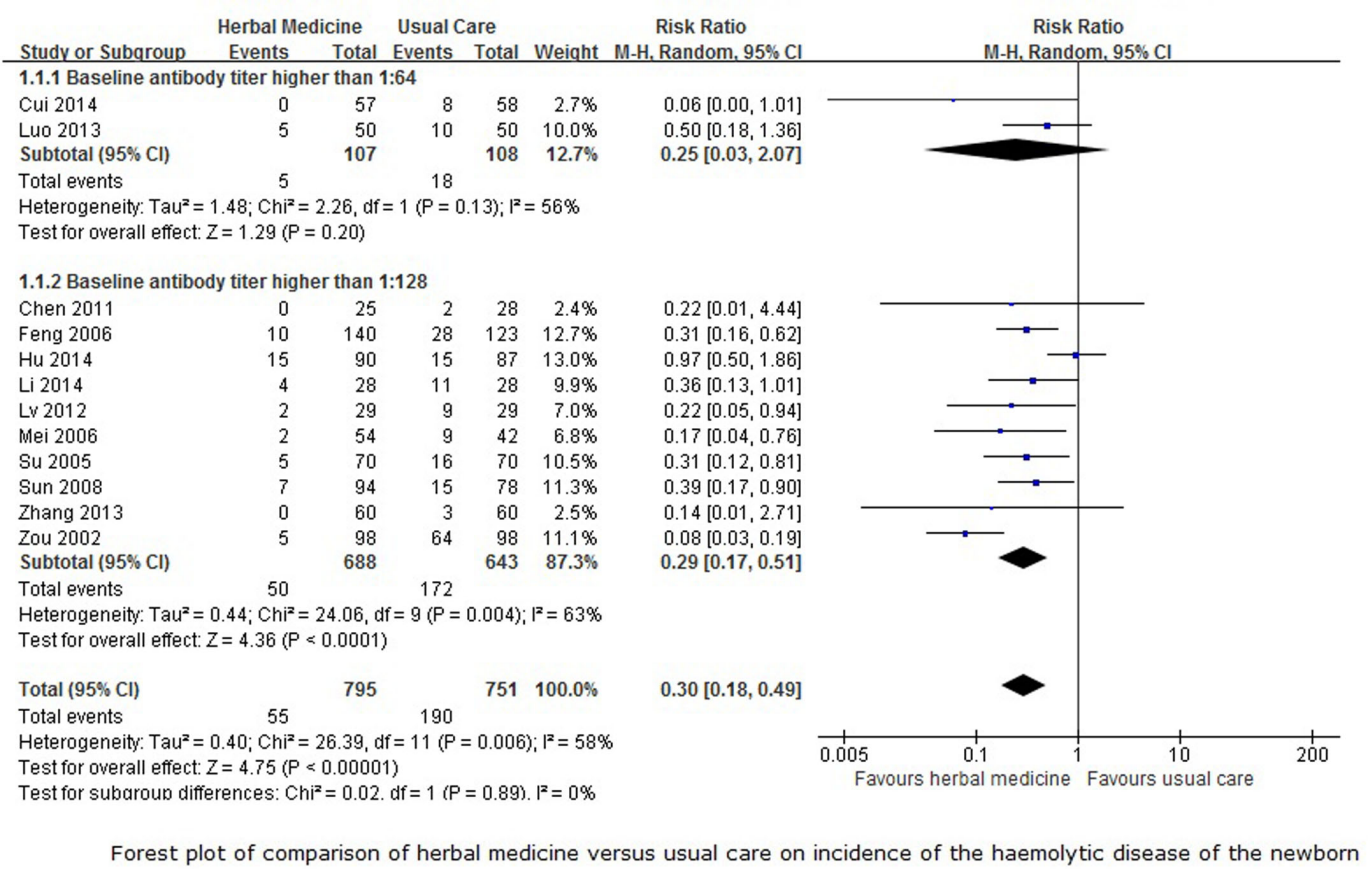
Forest plot for incidence rate of HDN.

**Fig 4:**
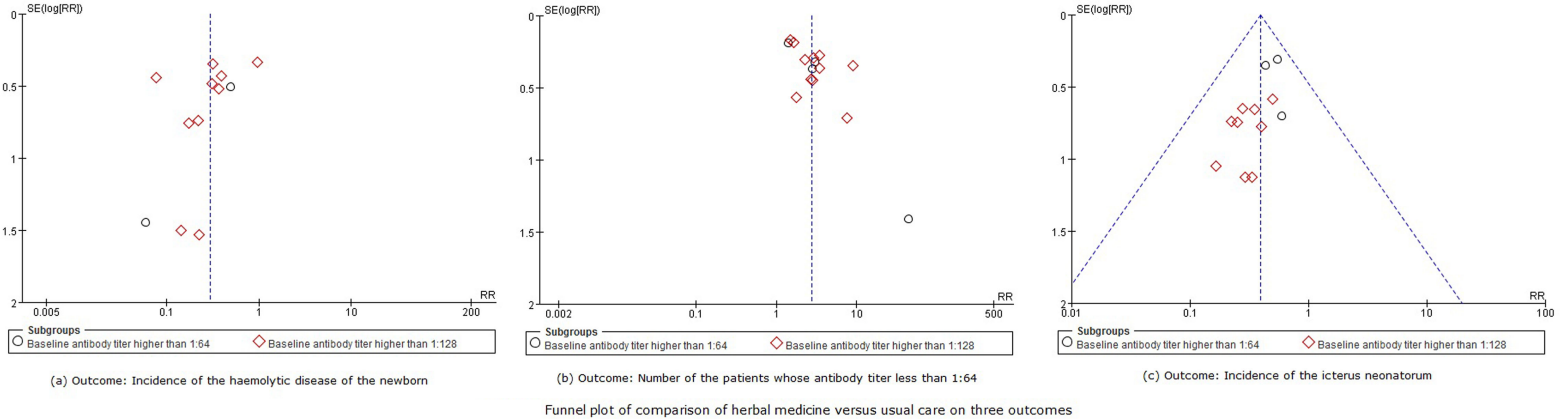
Funnel plot for three outcomes.

Data from twelve trials that reported the rate of HDN and compared CHM with usual care were pooled in a meta-analysis. The baseline antibody titer in two of the trials[20, 29] were higher than 1:64, and in the other ten trials [19, 22, 23, 26, 30, 31, 33, 36, 42, 46] were higher than 1:128, thus we performed subgroup meta-analyses on these 12 trials. With potential statistical heterogeneity between trials (50%< *I*^*2*^<75%), the subgroup analysis of participants with a baseline antibody titer that was higher than 1:64showed no difference between herbal medicine and usual care in decreasing the incidence of the HDN (RR 0.25, 95%CI 0.03 to 2.07, 215 participants, 2 trials, *I*^*2*^ = 56%, random-effect model, p = 0.20), however, herbal medicine may superior to the usual care when the baseline antibody titer of the participants was higher than 1:128 (RR 0.29, 95%CI 0.17 to 0.51, 1331 participants, 10 trials, *I*^*2*^ = 62%, random-effect model, p<0.0001). The overall meta-analysis (Fig 3) showed that CHM was better at reducing the incidence of HDN compared to usual care (RR 0.30, 95%CI 0.18 to 0.49, 1546 participants, 13 trials, *I*^*2*^ = 58%, random-effect model, p<0.00001). The funnel plot for this comparison showed considerable asymmetry (Fig 4A), which indicates that publication bias cannot be ruled out for this outcome.

One trial [25] also compared CHM with usual care and reported that no case of HDN occurred. One other trial [41] found CHM was superior to no treatment in reducing the number of HDN (RR 0.36, 95%CI 0.14 to 0.87, 170 participants). Another trial [16] reported no difference between herbal medicine plus usual care and usual care alone in reducing the incidence of the HDN when the baseline antibody titer of participants was higher than 1:128 (RR 0.42, 95%CI 0.17 to 1.04, 60 participants).

#### Secondary outcomes

Antibody titer after treatment

**Fig 5:**
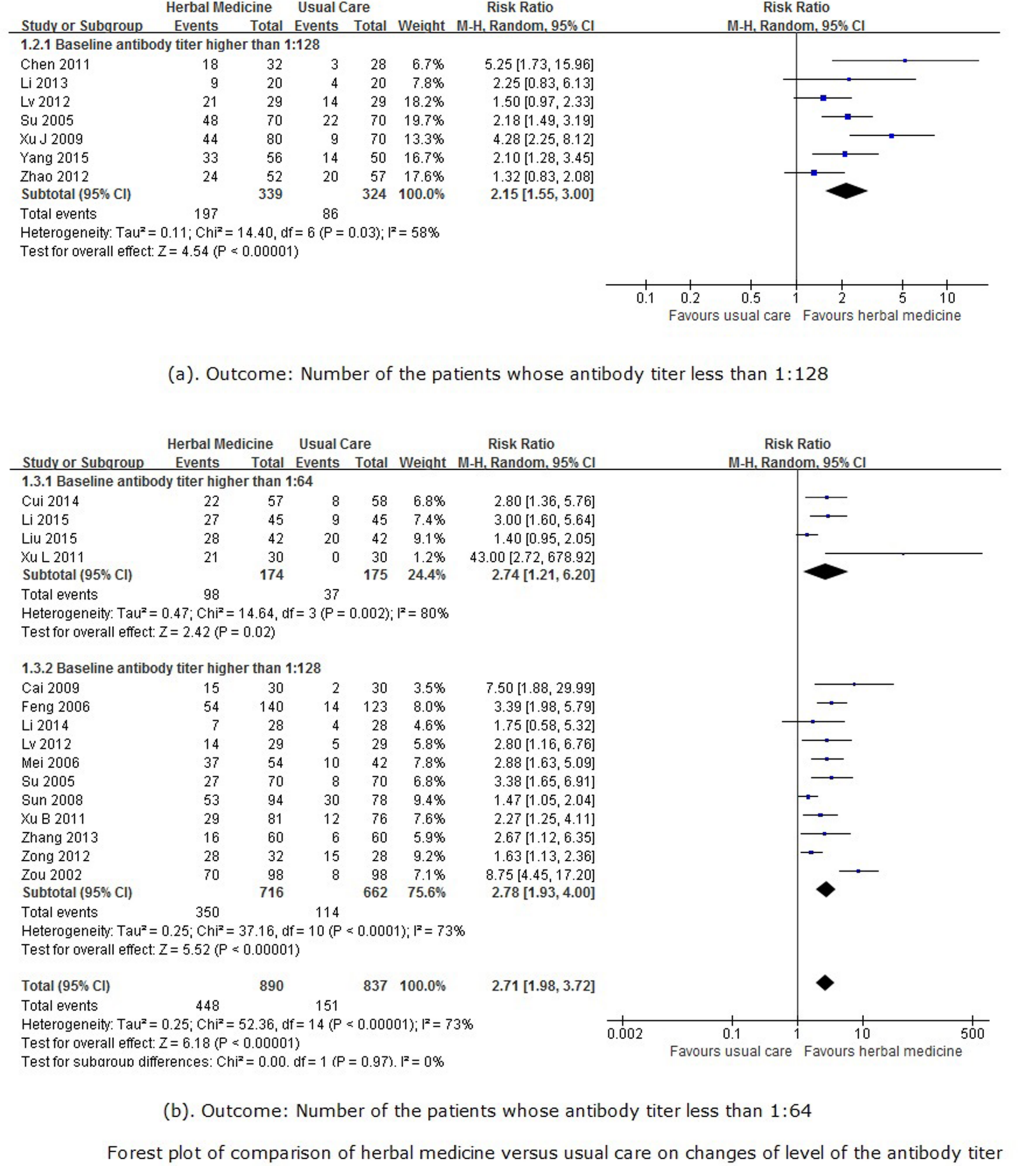
Forest plot for antibody titer value.

Seven trials [19, 25, 30, 33, 38, 40, 43] reported the number of patients with post-treatment antibody titers of less than 1:128. The meta-analysis of these studies (Fig 5A) showed that herbal medicine was superior to usual care in reducing the antibody titer to below 1:128(RR 2.15, 95%CI 1.55 to 3.00, 663 participants, 7 trials, *I*^*2*^ = 58%, random-effect model, p<0.00001). Another trial [24] found herbal medicine combined with usual care was superior to usual care alone for reducing antibody titers to below 1:128 (RR 3.14, 95%CI 1.59 to 6.23, 60 participants).

Another fifteen trials [18, 20–23, 26, 27, 29, 31, 36, 37, 39, 42, 45, 46] reported the number of patients with post-treatment antibody titers of less than 1:64.In the subgroup meta-analysis, herbal medicine was superior to usual care in reducing antibody titers to below 1:64 (RR 2.71, 95%CI 1.98 to 3.72, 1727 participants, 15 trials, *I*^*2*^ = 73%, random-effect model, p<0.00001), regardless of whether the baseline antibody titer was higher than 1:64 (RR 2.74, 95%CI 1.21 to 6.20, 349 participants, 4 trials, *I*^*2*^ = 80%, random-effect model, p = 0.02) or 1:128 (RR 2.78, 95%CI 1.93 to 4.00, 1378 participants, 11 trials, *I*^*2*^ = 73%, random-effect model, p<0.00001) (Fig 5B). There was also evidence of considerable asymmetry in the funnel plot (Fig 4B) for this comparison. The other two trials [28, 44] found there were no difference between herbal medicine plus usual care and usual care alone (RR 1.62, 95%CI 1.09 to 2.39, 161 participants, 2 trials, *I*^*2*^ = 27%, random-effect model, p = 0.02).

Incidence of the neonatal jaundice

**Fig 6:**
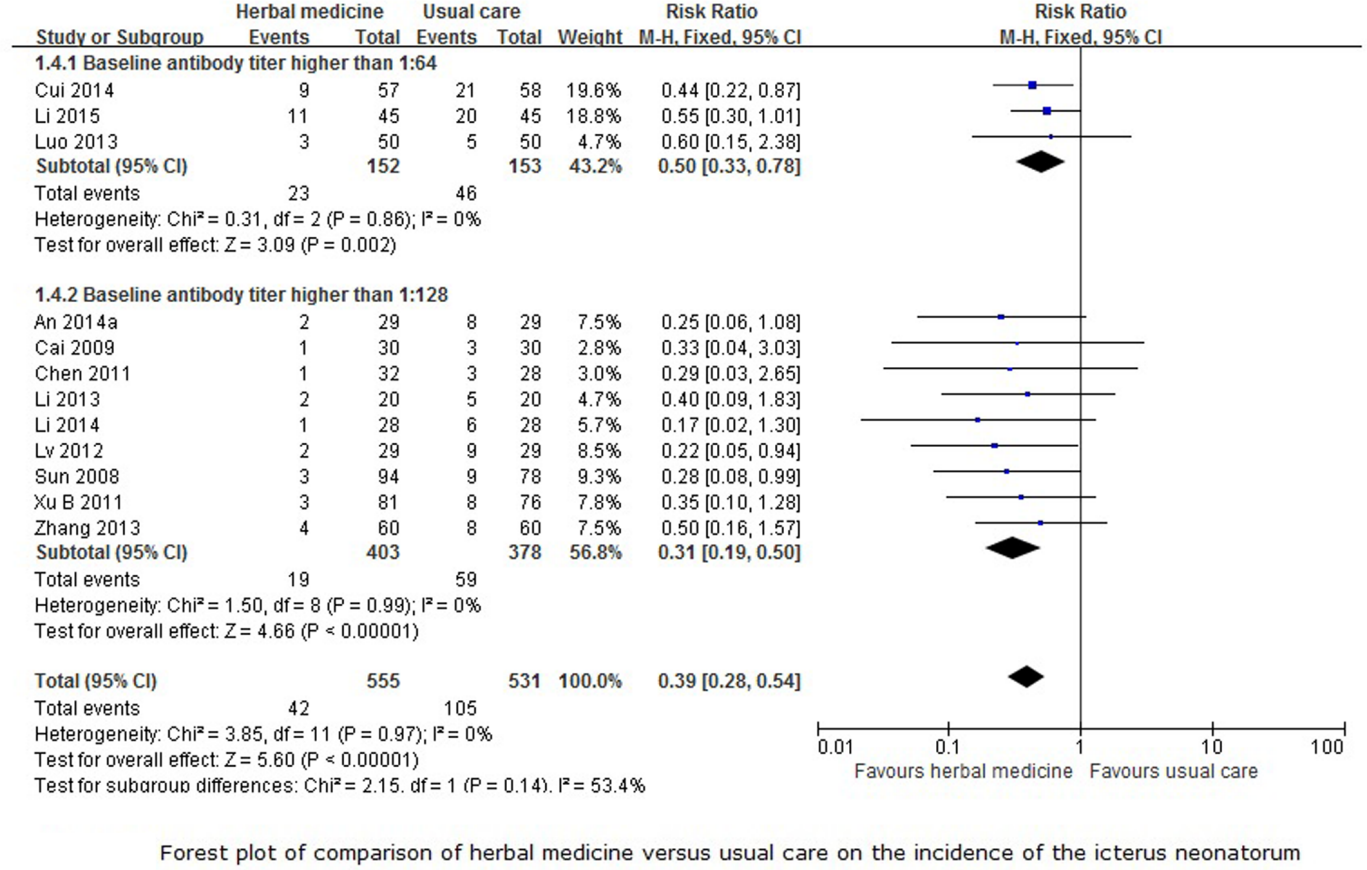
Forest plot for incidence of neonatal jaundice.

Twelve trials [15, 18–20, 25–27, 29, 30, 36, 37, 42] reported the incidence of the neonatal jaundice of the newborn with comparison between herbal medicine and usual care. The subgroup analysis showed that herbal medicine was more effective at reducing the incidence of neonatal jaundice compared to usual care whether the baseline antibody titer was higher than 1:64 (RR 0.50, 95%CI 0.33 to 0.78, 305 participants, 3 trials, *I*^*2*^ = 0%, fixed-effect model, p = 0.002) or 1:128 (RR 0.31, 95%CI 0.19 to 0.50, 781 participants, 9 trials, *I*^*2*^ = 0%, fixed-effect model, p<0.0001). The overall meta-analysis (Fig 6) also showed the same results (RR 0.39, 95%CI 0.28 to 0.54, 1086 participants, 12 trials, *I*^*2*^ = 0%, fixed-effect model, p<0.00001) for this outcome. The funnel plot for this comparison also showed considerable asymmetry (Fig 4C).

Another trial [16] reported no difference between herbal medicine plus usual care and usual care alone on reducing the incidence of neonatal jaundice when the baseline antibody titer of participants was higher than 1:128 (RR 0.25, 95%CI 0.06 to 1.08, 60 participants).

Umbilical cord blood bilirubin

One trial [20] where participants’ antibody titers were higher than 1:64 before treatment reported that herbal medicine may be superior to usual care in reducing umbilical cord blood bilirubin (MD -5.40 umol/L, 95%CI -7.76 umol/L to -3.04 umol/L, 115 participants). Similar results were reported in another five trials [18, 19, 26, 36, 38] where the baseline antibody titer of participants was higher than 1:128 (MD -4.05 umol/L, 95%CI -5.81 umol/L to -2.29 umol/L, 493 participants, 5 trials, *I*^*2*^ = 33%, random-effect model, p<0.00001). The overall meta-analysis showed that herbal medicine was superior to usual care in reducing unbilical cord blood bilirubin (MD -4.33 umol/L, 95%CI -5.84 umol/L to -2.82 umol/L, 608 participants, 6 trials, *I*^*2*^ = 35%, random-effect model, p<0.00001).

Another trial [16] reported an even larger effect when comparing herbal medicine as add-on compared with usual care alone (MD -10.10 umol/L, 95%CI -10.53 umol/L to -8.67 umol/L, 60 participants).

Apgar scores

There was no difference in the newborn’s Apgar scores between the herbal medicine and usual care groups in six trials [18–21, 23, 26, 36, 40] (MD 0.10, 95%CI -0.06 to 0.26, 572 participants, 6 trials, *I*^*2*^ = 0%, fixed-effect model, p = 0.24), irrespective of whether the baseline antibody titer was higher than 1:64 (MD 0.10, 95%CI -0.08 to 0.28, 457 participants, 5 trials, *I*^*2*^ = 0%, fixed-effect model, p = 0.29) or 1:128 (MD 0.10, 95%CI -0.27 to 0.47, 115 participants).

Another two trials [21, 23] only reported the numbers of the newborn with Apgar scores of less than 8, with no difference between the CHM and usual care groups (RR 0.97, 95%CI 0.29 to 3.22, 177 participants) and between CHM plus usual care and usual care alone (RR 0.67, 95%CI 0.12 to 3.82, 98 participants).

Birthweight

Five trials [18, 19, 23, 36, 40] reported the birthweight of the newborn, with no difference between the herbal medicine and usual care groups demonstrated in the meta-analysis (MD 0.06kg, 95%CI -0.04kg to 0.15kg, 578 participants, 5 trials, *I*^*2*^ = 41%, random-effect model, p = 0.27). Another trial [21] also found no difference in birthweight between herbal medicine plus usual care and usual care alone groups (MD 0.05kg, 95%CI -0.42kg to 0.52kg, 98 participants).

Adverse events

Twenty of the 28 included trials did not report whether there were any adverse events during the treatment period. Six [21, 23, 30, 38, 41, 42] of the remaining eight trials found no adverse events in herbal medicine group. Only two trials [16, 37] mentioned the adverse events which were present in both herbal medicine and control groups. One reported a case (3.33%) of abortion/stillbirth in herbal group and four cases (13.33%) of abortion/stillbirth in control group, and the other trial reported two cases (2.17%) of abortion/stillbirth in herbal group and ten cases (11.11%) of abortion/stillbirth in control group.

For the meta-analyses in which the fix-effects model and random effects model were both used as sensitive analysis, the results were consistent irrespective of potential heterogeneity between trials. Only the results of the meta-analysis using the random-effects model were reported in this review.

## Discussion

### Summary of main results

This review included 28 randomized controlled trials which assessed the effects of herbal medicine for preventing hemolytic disease of the newborn in pregnant women with ABO incompatibility and antibody titers that were higher than 1:64. The majority of the trials had unclear or high risk of bias according to the mentioned assessment criteria. The main results found that the rate of HDN and the incidence of neonatal jaundice in herbal medicine group might be 70% lower compared than in the usual care group (25.3% in usual care group, RR from 0.25 to 0.30); twice as many women taking herbal medicine (RR from 2.15 to 3.14) had post-treatment antibody titers lower than 1:64 compared with those receiving usual care, and umbilical cord blood bilirubin in herbal medicine group was 4 umol/L less than in the usual care group. There was no difference in Apgar scores and birthweights between groups. However, considering the poor methodological quality of the included trials, there remains some uncertainty about whether Chinese herbal medicine prevents HDN due to the ABO incompatibility.

Only a few included trials mentioned adverse events, with six trials reporting no adverse event in the herbal medicine group, and another two trials reported lower rates of fetal loss in the herbal medicine group (3%) than in the control group (13%). Due to insufficient information regarding the safety of herbal medicine for pregnant women, more trials will be needed to confirm its safety.

### Overall completeness and applicability of evidence

We searched two English databases and four Chinese databases without language restrictions. However, all of the 28 included trials from this review were conducted and published in China. In countries outside of China, there is currently no standard therapy for treating maternal-fetal ABO incompatibility during pregnancy, with therapeutic procedures only being provided if the newborn was diagnosed with HDN after birth. Thus, the results of this review may have significant implications for providing a preventative therapy for HDN caused by ABO incompatibility.

### Quality of the evidence

As previously mentioned, only five trials were evaluated as having low risk of bias. The remaining 23 trials all have poor methodology and have high or unclear risk of selection, attrition, reporting and other bias.

We employed the Grading of Recommendations Assessment, Development and Evaluation (GRADE) criteria ratings to assess the quality of the evidence; factors that downgraded the quality include imprecision, inconsistency, indirectness, limitations and bias of the evidence. For the meta-analyses in this review, we downgraded the level of evidence mainly because of the potential for performance, attrition, and reporting bias and the inconsistency of the results between the included studies. Subsequently, we could find only very low-quality evidence that CHM may reduce the incidence of HDN and reduce antibody titers, and low-quality evidence that CHM may reduce the incidence of neonatal jaundice and umbilical cord blood bilirubin levels (Table 3 Summary of finding table).

**Table 3:** Summary finding table with herbal medicine vs. usual care for maternal and infant blood type incompatibility.

**Patient or population:** Pregnant women with maternal and infant blood type incompatibility					
**Settings:** The outpatient department of the traditional Chinese medicine hospital					
**Intervention:** Herbal medicine versus usual care					
**Outcomes**	Illustrative comparative risks* **(95% CI)**		**Relative effect**	**No of Participants**	**Quality of the evidence**
	Assumed risk	Corresponding risk			
			**(95% CI)**		**(GRADE)**
	**Usual care**	**Chinese herbal medicine**			
**Incidence of the hemolytic disease of the newborn**	**253 per 1000**	**76 per 1000**	**RR 0.3**	1546	⊕⊝⊝⊝
		(46 to 124)	(0.18 to 0.49)	(12 studies)	**very low**^1^^,^^2^^,^^3^
**Number of the patients whose antibody titer less than 1:128**	**265 per 1000**	**571 per 1000**	**RR 2.15**	663	⊕⊝⊝⊝
		(411 to 796)	(1.55 to 3)	(7 studies)	**very low**^1^^,^^2^^,^^3^
**Number of the patients whose antibody titer less than 1:64**	**180 per 1000**	**489 per 1000**	**RR 2.71**	1727s)	⊕⊝⊝⊝
		(357 to 671)	(1.98 to 3.72)	(15 studies	**very low**^1^^,^^3^^,^^4^
**Incidence of the neonatal jaundice**	**198 per 1000**	**77 per 1000**	**RR 0.39**	1086	⊕⊕⊝⊝
		(55 to 107)	(0.28 to 0.54)	(12 studies)	**low**^1^^,^^3^
**Umbilical cord blood bilirubin (umol/L)**	The mean umbilical cord blood bilirubin (umol/l) in the control groups was**32.35 umol/L**	The mean umbilical cord blood bilirubin (umol/l) in the intervention groups was**4.33 lower**(5.84 to 2.82 lower)		608	⊕⊕⊝⊝
				(6 studies)	**low**^1^^,^^3^
**Apgar scores**(Scale from0 to 10)	The mean Apgar scores in the control groups was**9.23**	The mean Apgar scores in the intervention groups was**0.1 higher**(0.06 lower to 0.26 higher)		572	⊕⊕⊝⊝
				(6 studies)	**low**^1^^,^^3^
**Weigh of newborn (kg)**	The mean Birthweight in the control groups was**3.10**	The mean Birthweight in the intervention groups was**0.06 higher**(0.04 lower to 0.15 higher)		578	⊕⊝⊝⊝
				(5 studies)	**very low**^1^^,^^2^^,^^3^

*The **corresponding risk** (and its 95% confidence interval) is based on the **assumed risk** in the comparison group and the **relative effect** of the intervention (and its 95% CI).

**CI:** Confidence interval; **RR:** Risk ratio

GRADE Working Group grades of evidence

**High quality:** Further research is very unlikely to change our confidence in the estimate of effect.

**Moderate quality:** Further research is likely to have an important impact on our confidence in the estimate of effect and may change the estimate.

**Low quality:** Further research is very likely to have an important impact on our confidence in the estimate of effect and is likely to change the estimate.

**Very low quality:** We are very uncertain about the estimate.

^1^ Majority trials had unclear risk of selection, detection, attrition and reporting bias

^2^ The I square test showed a significant statistical heterogeneity among trials (I-square larger than 50%)

^3^ The asymmetric funnel plot indicated the possibility of publication bias.

^4^ The I square test showed a significant large statistical heterogeneity among trials (I square larger than 70%)

### Agreements and disagreements with other studies or reviews

Two other reviews [1, 48] were found on this topic, which were all dissertations for masters’ degrees and written in Chinese, with one of them being published in 2015 [1]. One review included eleven trials and the other included 20 trials. Both of them used a total effective rate as the primary outcome measure, which is a composite outcome index combining the reduction of antibody titers, rate of HDN and adverse birth outcomes post treatment. Although this outcome index is commonly used in China, it is difficult to determine its clinical relevance since the definition of “effectiveness” is inconsistency between studies. These two reviews also conducted meta-analyses to investigate the effects of CHM in reducing the rate of HDN, with results consistent with our studies, showing that CHM was more likely to reduce the incidence of HDN compared to usual care (RR = 0.19 or 0.25, p<0.05). One of the reviews also reported there was no difference between CHM and usual care in Apgar scores and birthweight.

Our review included 28 trials and assessed the effects of CHM for reducing the incidence of HDN, the antibody-titer, the incidence of neonatal jaundice and umbilical cord blood bilirubin. Subgroup-meta analyses were conducted for the participants with different baseline antibody titer values. We found similar results to the previous reviews, showing that CHM is effective in reducing the incidence of HDN (RR = 0.30, p<0.05), with the overall quality of the evidence appraised according to the GRADE criteria.

### Implications for practice

Maternal-fetal ABO incompatibility may be asymptomatic during pregnancy, and patients may not be aware they have this condition. As there are currently no guidelines or standard therapies to prevent HDN caused by ABO incompatibility during pregnancy, this study may have identified a possible therapy for preventing this condition.

According to the review, five herbs are frequently used to prevent HDN caused by maternal-fetal ABO incompatibility, including Herba Artemisiae Scopariae (Yin Chen), Rhizoma Glycyrrhizae (Gan Cao), Fructus Gardeniae (Zhi Zi), Radix Scutellariae (Huang Qin), and Radix et Rhizoma Rhei (Da Huang). Among these five herbs, Yin Chen has been commonly used for treating jaundice and HDN.A laboratory study [49] found that liver fibrosis induced by dimethylnitrosamine in rats was improved by the administration of Yinchenhao Decoction (in which Yin Chen was the primary active ingredient), with a reduction in hydroxyproline and a significant improvement in liver function and hepatic histology after 2 weeks of treatment. Da Huang was found to promote the secretion of bile, causing gallbladder and biliary sphincter relaxation which may relieve jaundice. The potential mechanism may be through increased expression of transport proteins associated with the metabolism of bile acids to reduce the accumulation of bile acids and other toxic compounds in the liver [50, 51], which could improve the ultrastructure of liver cells, thus affecting the intracellular enzyme activity and the concentration of calcium ions in the cells [52]. Huang Qin is thought to be a heat-clearing, phlegm-removing herb, traditionally used to cool heat, clear damp-heat, and calm the fetus [53]. It is also found to contain compounds with anti-inflammatory and antimicrobial effects [54].Zhi Zi has effects on the contraction of gallbladder, containing iridoid and Saffron glucoside which may increase the secretion of bile [55].Even though there are some studies (cited as above) tried to investigate the potential mechanism of the specific herbs in treating HDN, we need to aware that the mechanisms of how the Chinese herbal medicine could eliminate maternal IgG antibodies were not evidenced.

Regarding safety considerations, medications used during pregnancy require extra caution. According to Chinese herbal medicine traditions, herbs that work on clearing heat are usually forbidden during pregnancy, as it is believed that oral intakes of these herbs may cause abortions and other serious adverse birth outcomes. However, four of the top five most frequently used herbs in the included trials from this review are herbs that have the function of clearing heat [53]. The majority of trial participants took the herbal decoction for at least half of their pregnancy until labor, with no or less adverse events (such as fetal loss) reported in the treatment group compared with the usual care group. This finding suggests that it may be safe for pregnant women with ABO incompatibility to use CHM with herbs that have the function of clearing heat and draining dampness, under the guidance of herbal medicine practitioners. According to our study, the incidence of HDN from ABO incompatibility may decrease from 25.3% to 7.6% when CHM is used during pregnancy, compared to usual care. However, we have low confidence in certainty of the safety issue using Chinese herbal medicine to prevent or treat ABO HDN based on the current evidence.

## Conclusions

This review of28 included trials found very low-quality evidence that CHM using herbs with the function of clearing heat and draining dampness may prevent hemolytic disease of the newborn when used in pregnant women with ABO incompatibility. Due to insufficient information, no firm conclusions can be draw based on this review’s results regarding the effectiveness of CHM for this condition, as well as the safety of the relevant herbal medicine for pregnant women.

## Supporting information

S1 File The preliminary protocol of the review. (PDF)

S2 File PRISMA checklist. (DOCX)
