# Supplementary material for: Oral administration of Chinese herbal medicine during gestation period for preventing hemolytic disease of the newborn due to ABO incompatibility: A systematic review of randomized controlled trials
Source: PLoS One. 2017 Jul 18;12(7):e0180746. doi: 10.1371/journal.pone.0180746 (PMC5515403; doi:10.1371/journal.pone.0180746)
Supplement: S1 File — (PDF) [file pone.0180746.s001.pdf]

PROSPERO International prospective register of systematic reviews

Oral administration of Chinese herbal medicine during the gestation period for preventing hemolytic disease of the newborn due to ABO incompatibility: a systematic review of randomized controlled trials

Huijuan Cao, Ruohan Wu, Mei Han, Jianping Liu

Citation

Huijuan Cao, Ruohan Wu, Mei Han, Jianping Liu. Oral administration of Chinese herbal medicine during the gestation period for preventing hemolytic disease of the newborn due to ABO incompatibility: a systematic review of randomized controlled trials. PROSPERO 2016:CRD42016038637 Available from
http://www.crd.york.ac.uk/PROSPERO_REBRANDING/display_record.asp?ID=CRD42016038637

Review question(s)

Does the oral administration of Chinese herbal medicine during the gestation period reduce the incidence rate of ABO incompatibility hemolytic disease of the newborn?

Searches

Two English databases and four Chinese databases will be searched from inception to May 2016, including the Cochrane Central Register of Controlled Trials (CENTRAL), PubMed, China Network Knowledge Infrastructure (CNKI), Chinese Scientific Journals Database (VIP), WanFang Database (for unpublished graduate theses in China), and Chinese Biomedicine (CBM).

Details of the search strategies are shown as below.

#1: Search "Medicine, Chinese Traditional" [Mesh]; #2:Search "Drugs, Chinese Herbal" [Mesh]; #3: Search "ABO incompatibility" [Mesh]; #4:Search “ABO hemolytic disease of newborn” [Mesh]; #5: Search (#1 OR #2) AND (#3 OR #4)

The above strategies will be adopted for each specific database, the Chinese characters for relevant key words will be used when searching Chinese databases.

Types of study to be included

Randomized controlled trials.

Condition or domain being studied

The maternal and infant ABO type incompatibility.

Participants/ population

The pregnant woman whose blood type is "O", and the husband whose blood type is A, B or AB; and an antibody titer (IgG anti-A or anti-B) higher than 1:64.

Intervention(s), exposure(s)

The intervention is to include any type of oral administration of Chinese herbal medicine, regardless of the formula or ingredients.

Comparator(s)/ control

The control could be no treatment, placebo, usual care or standard treatment for the condition. A combination of Chinese herbal medicine and other therapies compared to other therapies will also be included.

Outcome(s)

Primary outcomes

Incidence rate of hemolytic disease of the newborn.

Secondary outcomes

The antibody titer after treatment;

The incidence rate of the icterus neonatorum;

Neonatal bilirubin (including neonatal umbilical cord blood bilirubin and total bilirubin);

Other measurements for health status of the newborn (such as Apgar scores, weight of the newborn, etc.);

Adverse events.

Data extraction, (selection and coding)

Two review authors (Cao HJ and Wu RH) will independently assess the titles, abstracts and keywords of every record retrieved in terms of relevance and design according to the selection criteria. Full articles will be retrieved for further assessment against the inclusion criteria; reasons for those that are screened out during this phase will be recorded. Where differences in opinion exist, they will be resolved by discussion until a consensus is reached.

Data concerning details of the included studies will be extracted independently by two review authors (Cao HJ and Wu RH) using a piloted data extraction form. The data extraction form will include the general information, the characteristics of the study design, the participants, intervention and outcomes. Authors of relevant studies identified will be contacted if needed in order to obtain additional references, unpublished trials, or data missing from the original trials. Disagreements will be resolved by consensus.

Risk of bias (quality) assessment

Two authors (Cao HJ and Han M) will assess the methodological quality of the included trials independently. Selection bias (random sequence generation and allocation concealment), detection bias (blinding of outcome assessment), attrition bias (incomplete outcome data), reporting bias (selective reporting), and other biases (determined according to sample size calculation method, inclusion/exclusion of criteria for patients' recruitment, comparability of baseline data, funding sources, and any other potential methodological flaw that might have influenced the overall assessment) will be assessed according to the criteria from the Cochrane Handbook for Systematic Reviews of Intervention. Since all of the outcomes of this review will be measured objectively, and are unlikely to be influenced by loss of blinding method; and there is no ideal placebo control for Chinese herbal medicine (especially for the herbal decoction), we will not assess the performance bias with blinding of participants and personnel. Three potential bias judgments: low risk, high risk, and unclear risk, will be determined for each single trial during assessment. A judgment of low risk will be made when all the seven items meet the criteria as “low risk”, a judgment of high risk of bias will be made when at least one of the seven items is assessed as “high risk”.

Strategy for data synthesis

Data will be summarized using risk ratio (RR) with 95% confidence intervals (CI) for binary outcomes or mean difference (MD) with 95% CI for continuous outcomes. We will use RevMan 5.3 software from the Cochrane Collaboration for data analyses. Meta-analysis will be conducted if the trials have an acceptable homogeneity on study design, participants, interventions, control, and outcome measures. Statistical heterogeneity is tested by examining I-squared, meaning that an I-squared larger than 50% indicates the possibility of statistical heterogeneity. Both fixed effect modelling and random effects modelling will be used if there is a possibility of statistical heterogeneity among trials. Pooling analysis will not be done if there is an obvious huge statistical heterogeneity (I-squared>75%). Publication bias will be explored by funnel plot analysis.

Analysis of subgroups or subsets

Subgroup analyses will be conducted to determine the evidence for the inconsistent baseline characteristics or different treatment duration if data is sufficient. Sensitivity analyses will be used in order to determine whether the conclusions differed if eligibility is restricted to studies without high risk of bias; or if a fixed effect/random effect model have been applied.

Contact details for further information

Huijuan Cao

88 mailbox, Beijing University of Chinese Medicine,

Bei San Huan Dong Lu 11,

Chaoyang District, Beijing, 100029,

China

huijuancao327@hotmail.com

Organisational affiliation of the review
Beijing University of Chinese Medicine

Review team
Dr Huijuan Cao, Beijing University of Chinese Medicine
Dr Ruohan Wu, Beijing University of Chinese Medicine
Dr Mei Han, Beijing University of Chinese Medicine
Professor Jianping Liu, Beijing University of Chinese Medicine

Anticipated or actual start date
15 May 2016

Anticipated completion date
31 July 2016

Funding sources/sponsors
Beijing Municipal Organization Department Talents Project

Conflicts of interest
None known

Language
English

Country
China

Subject index terms status
Subject indexing assigned by CRD

Subject index terms
Administration, Oral; Blood Group Incompatibility; Complementary Therapies; Drugs, Chinese Herbal; Hematologic Diseases; Humans; Infant Health; Infant, Newborn; Infant, Newborn, Diseases; Pregnancy; Treatment Outcome

Stage of review
Ongoing

Date of registration in PROSPERO
03 May 2016

Date of publication of this revision
03 May 2016

Stage of review at time of this submission	Started	Completed
Preliminary searches	Yes	No
Piloting of the study selection process	No	No
Formal screening of search results against eligibility criteria	No	No
Data extraction	No	No
Risk of bias (quality) assessment	No	No
Data analysis	No	No

PROSPERO
International prospective register of systematic reviews
The information in this record has been provided by the named contact for this review. CRD has accepted this information in good faith and registered the review in PROSPERO. CRD bears no responsibility or liability for the content of this registration record, any associated files or external websites.
